# Editorial: Color Vision Sensation and Perception

**DOI:** 10.3389/fpsyg.2016.01084

**Published:** 2016-07-19

**Authors:** Marcelo F. Costa

**Affiliations:** ^1^Departamento de Psicologia Experimental, Instituto de Psicologia, Universidade de São PauloSão Paulo, Brasil; ^2^Núcleo de Neurociências e Comportamento e Neurociências Aplicada, Universidade de São PauloSão Paulo, Brasil

**Keywords:** color discrimination, congenital color blindness, luminance, mesopic vision, scotopic vision, clinical psychophysics, perceptual organization, color naming

Color vision is one of the most intriguing phenomena of the visual experience and has been the object of study from vision scientists to philosophers of perception. The wavelengths, their intensity and purity are experienced as hue, brightness and saturation based on complex information processing of the light entering the eyes. This special research topic is devoted to covering from basic aspects based on light and retinal processes to perceptual and cognitive mechanisms and their respective applications in clinical settings.

The reader will find a series of papers organized in two chapters, one devoted to the more basic phenomena and processing, the other focused on the more cognitive and cultural aspects.

The integrative aspect of luminance in color vision starts our journey. Reduction in number of levels of luminance in pseudoisochromatic stimulations affects the chromaticity thresholds measured psychophysically, suggesting interactions between luminance and color in those stimuli with luminance steps lower than seven (Souza et al.). Color sensation was also investigated under low-light levels. Mesopic and scotopic conditions could be considered an interference zone for color vision since rods activities generate physiological interferences in cone-driven retinal chromatic pathways. This review paper keeps us up to date regarding the range and impact of rod–cone interactions on human visual function and performance (Zele and Cao).

Clinical applications centered on congenital color blindness end the first chapter. Sensory psychophysical thresholds on the tritanopic color confusion axis were investigated in protanomalous and deuteranomalous subjects (Costa et al.). Reduction in discrimination was found for both types of congenital color blindness, protanomalous and deuteranomalous, but subjects in the latter group showed worse results. Electrophysiological assessments regarding luminance and compound stimuli (luminance plus red-green stimulus) were also evaluated in congenital color blindness subjects. Since compound stimuli elicited small or no response in red-green congenital color blinds, this finding could indicate that the cortical response for compound stimuli in the present experiment was dominated by chromatic contribution (Risuenho et al.).

A search for discrimination thresholds addressed using the pseudoisochromatic plates configures a new psychophysical application for the traditional and widely used color test (Jurasevska et al.).

Basic perceptual phenomena of Gestalt theories regarding color vision were addressed using figure-ground perception on saturation and contrast polarity modulations. The study conducted by Dresp-Langley and Reeves investigated the lightness dependency of the achromatic loci in color space. The results pointed toward a hitherto undocumented functional role of color saturation in the genesis of form, and in particular figure-ground percepts. Two experiments conducted by Kuriki showed that first, a color-appearance space normalized to daylight may be defined for the human visual system and second, that color space could reduce the discrepancy between the achromatic loci and the lightness axis of the color appearance models. The last study of the perceptual mechanism section relates retinal image quality for the display red and blue pixel radiation with the chromostereopsis retinal disparity achieved (zolinsh and Muizniece).

Color is also important from an anthropological view in which a long debate regarding the existence of natural categories of hue and their universality has been addressed by ethological, electrophysiological, behavioral and psychophysical perspectives. The color naming and gender section presents contributions regarding the variations in color naming occurring in congenital color blindness subjects and the gender and cultural aspects related to color preferences. The study of Nagy et al. shows that dichromatic subjects involve the brightness properties of the different spectral stimuli when judging their chromatic content and that at the shorter wavelengths the signal of the intact tritos photoreceptor dominates the decision making in color naming tasks, even for the anopes. Al-Rasheed provides evidence that both sex differences and cultural differences are relevant in hue preference in terms of how stimulus-background cone-contrast was weighted summarizing color preference quantitatively rather than using subjective color names.

The question of how we see colors and how we can discuss their impact in many dimensions of our life is an actual and multidiscipline inquiry to which we hope to contribute with this special research topic.

Enjoy this special topic on color vision.

## Author contributions

The author confirms being the sole contributor of this work and approved it for publication.

## Funding

Supported by Conselho Nacional de Desenvolvimento Científico Edital Universal 440357/2014-4. MFC is a level 2 research fellow.

### Conflict of Interest Statement

The author declares that the research was conducted in the absence of any commercial or financial relationships that could be construed as a potential conflict of interest.

